# The association between family support and college students’ well-being: an analysis of mediating pathways

**DOI:** 10.3389/fpsyg.2026.1860654

**Published:** 2026-09-18

**Authors:** Mengyao Chai, Shimin Su, Yixiao Wang, Jiaqi Shi, Qian Wang, Shuqing Zhou, Huiling Dong, Bingyi Wu, Tingting Xu

**Affiliations:** 1 School of Management, Shandong Second Medical University, Weifang, China; 2 College of Liberal Arts and Social Sciences, City University of Hong Kong, Kowloon, Hong Kong SAR, China; 3 Department of Nursing, The Affiliated Hospital of Shandong Second Medical University, Weifang, China; 4 School of Public Health, Shandong Second Medical University, Weifang, China; 5 School of Nursing, Shandong Second Medical University, Weifang, China

**Keywords:** chain mediation, family support, psychological capital, school support, university students, well-being

## Abstract

**Introduction:**

The issue of student mental health represents a key concern for higher education institutions. Their well-being is associated with multiple factors, including family environment and institutional education. This study aims to explore the relationship between family support and student well-being, with mediating roles of school support and psychological capital.

**Methods:**

This study employed a cross-sectional research design with a multi-stage stratified cluster sampling method to collect questionnaire data from 10,726 college students in Shandong Province, China. Core variables were measured using the Mental Health Continuum-Short Form (MHC-SF), the Family APGAR Index, the School Support Scale, and the Psychological Capital Scale. Independent samples *t*-tests and analysis of variance were used for comparative analyses, and multiple linear regression alongside structural equation modeling to investigate the associations between various factors and college students’ well-being.

**Results:**

Significant differences in well-being scores were observed across academic year, major, program level, family structure, and parental workplace, both mother and father, at *P* < 0.05 multiple linear regression analysis showed that psychological capital, family support, and school support were all significantly positively correlated with college students’ well-being scores. Among these, psychological capital was identified as a significant mediating variable in the model. Structural equation modeling (SEM) further revealed that the association between family support and well-being comprised both a direct path and indirect paths via school support and psychological capital.

**Discussion:**

Overall, for students with diverse family backgrounds, there is a significant direct association between family support and well-being. In addition, this association can be realized through three indirect pathways: (1) an independent mediating pathway via school support, (2) an independent mediating pathway via psychological capital, and (3) a sequential mediating pathway via both school support and psychological capital. It is important to note that the measurement of psychological capital in this study employed a simplified operationalization based on positive emotion items as a proxy indicator for cognitive psychological resources; however, this measurement approach has limitations in terms of construct coverage, and this should be carefully considered when interpreting the research findings.

## 1 Introduction

The student mental health crisis, exhibiting trends toward earlier manifestation and broader prevalence, is significantly driven by a lack of positive psychological resources and the inadequacy of social support systems. Mounting research evidence indicates that psychological problems in the student population have become a worldwide public health challenge. A research report based on the WHO World Mental Health Surveys, covering 1,572 college students across 21 countries, shows that the 12-month prevalence of mental disorders is 20.3%, with 83.1% of cases having onset prior to college entry (Auerbach et al., 2016). Another WHO-led international college student survey (WMH-ICS), conducted among 13,984 first-year students at 19 universities across 8 countries, further confirmed that at least one-third of the students screened positive for lifetime mental disorders (Auerbach et al., 2018). A recent national survey covering 41,620 students from 106 Chinese universities reached similar alarming conclusions: approximately 9.8% of the students screened positive for depressive symptoms, 11.5% for anxiety symptoms, and the depression-anxiety comorbidity rate was 6.5% (Han et al., 2025). These findings indicate that psychological problems are not only ubiquitous among university students globally but also frequently predate their enrollment, highlighting an alarming situation. Hence, systematically examining the pathways that influence student well-being is a critical endeavor of both theoretical and practical relevance.

Reflecting an individual’s integrated appraisal of life quality and meaning, well-being is an important and stable measure of mental health status. Family support is the first and most sustained source of social support in the developmental process of a child, occupying a pivotal position among the determinants of student well-being. Previous research has consistently demonstrated that family support not only directly influences college students’ well-being through emotional and material assistance but also exerts indirect effects through multiple mediating pathways (Xu et al., 2019). In the existing literature, family support is frequently regarded as a core independent variable. Scholars have employed statistical methods such as structural equation modeling (SEM) and hierarchical regression analysis to investigate how it influences the well-being of university students through mediating variables such as psychological capital and school support. Huang and Zhang (2022) demonstrated that during the pandemic, perceived social support (including family support) improved well-being and stress management by fostering psychological capital. Furthermore, Wang et al. (2023), through a two-wave longitudinal study, confirmed the serial mediating effect of psychological capital and problem-focused coping in the relationship between family support and well-being, emphasizing that the positive psychological resources developed in the family environment are the fundamental internal mechanism for sustaining well-being.

In addition, Butler et al. (2022) pointed out that school support may play a complementary or reinforcing role in the relationship between family support and well-being. They emphasized that a supportive school environment can serve as a critical protective factor, especially when family support is limited. In this research field, two typical pathways have emerged. The first is the direct pathway, which operates through specific emotional care, instrumental support, and value recognition, directly promoting students’ well-being (Ratelle et al., 2013). The second is indirect, operating either through the internal, resource-based mediation of psychological capital or the external, contextual mediation of school support (Luthans et al., 2010). Although existing studies have confirmed the robust associations of family, school support, and psychological capital with well-being, and theoretical frameworks suggest they constitute an interconnected support system, empirical evidence remains scarce regarding their synergistic mechanisms and integrative pathways. Furthermore, the potential influence of early structural factors, such as place of origin and family structure on these dynamics is yet to be examined.

Following are the theoretical frameworks and related research hypotheses this study will explore:

Family support is widely recognized as a critical factor that positively influences the well-being of university students. The family investment model posits that parental investment of economic and other resources promotes children’s cognitive and social development, thereby increasing their well-being (Deng et al., 2016). In contrast, the family stress model posits that family socioeconomic pressure negatively impacts children’s mental health indirectly, primarily by increasing familial conflict and reducing available resources. Economic stress may elicit parental psychological distress and marital conflict, thereby compromising the effectiveness of family support and ultimately diminishing the well-being of university students (Zhang et al., 2021). Based on this theoretical framework, the following research hypothesis is proposed:

H1: Family support positively predicts the well-being of college students.

Conservation of resources theory posits that individuals rich in resources are better safeguarded against loss and can leverage existing resources to gain more, initiating an upward spiral of resource accumulation (Hobfoll, 2001). Therefore, greater family support is associated with increased access to school support. Similarly, Yuen and Wu (2024) found that positive interactions and support from peers and teachers were significantly associated with higher levels of well-being. Based on this theoretical and empirical rationale, the present study proposes the following research hypothesis:

H2: School support plays a mediating role in the relationship between family support and university students’ psychological well-being.

According to psychological capital theory, external support positively contributes to the enhancement of an individual’s psychological capital (Luthans et al., 2015). Therefore, family support contributes to the building and enhancement of an individual’s psychological resources (Siu et al., 2014). This study further proposed that students with higher levels of psychological capital are more capable of positively interpreting and mobilizing the resources provided by family support. These resources are subsequently transformed into stronger academic motivation and adaptive behaviors, ultimately leading to enhanced subjective well-being. This process aligns with the core premise of conservation of resources theory, whereby individuals leverage existing resources to acquire new resources, creating a positive upward spiral (Hobfoll, 1989). In other words, family support may not only directly enhance an individual’s well-being but also indirectly influence it through the improvement of psychological capital. Therefore, the present study proposes the following research hypothesis:

H3: Psychological capital serves as a mediator between family support and student well-being.

Psychological Capital is a positive psychological state that individuals exhibit during their development, comprising four core components: hope, self-efficacy, resilience, and optimism. These four components together form a higher-order construct, collectively referred to as the “HERO” model (Newman et al., 2014). Specifically, self-efficacy refers to an individual’s confidence in their ability to successfully complete challenging tasks and to exert the necessary effort; hope refers to the willpower to persevere toward goals and the capacity to find alternative pathways when encountering obstacles; resilience refers to the ability to persist, bounce back, or even surpass previous levels in the face of difficulties, setbacks, and adversity; and optimism refers to a positive attributional style and positive expectations for success in the present and future (Liu et al., 2024). Students with strong school support typically possess higher psychological capital; in contrast, impoverished teacher-student interactions and a perceived lack of support are associated with lower levels (Liu, 2017). These findings suggest that students with strong family support gain access to greater school support, which in turn helps them build higher levels of psychological capital, thereby ultimately enhancing their overall well-being. Consequently, this study proposes the following hypothesis:

H4: School support and psychological capital play a chain-mediating role between family support and well-being.

Disparities in geographic origin exert a cascading influence on students’ well-being; the typically lower levels of material and emotional support available to rural students compared with their urban peers not only hinders the development of psychological capital but also directly reflects the observed gap in well-being levels (Ye et al., 2020). Family structure constitutes another key determinant. Research suggests that a single-parent family background can negatively impact subjective well-being by diminishing psychological capital, primarily through pathways such as a weakened perception of social support (Amato, 2010). Thus, our study proposes the following hypothesis:

H5: Lower levels of family support, psychological capital, and well-being are predicted among students from rural areas and single-parent families, relative to their urban counterparts and those from non-single-parent families.

This study employs SEM with a bootstrapping approach to examine both the direct and mediating effects of family/school support and psychological capital on well-being. In addition, this study included place of origin and family structure as exogenous variables in the analysis to control for the effects of demographic characteristics on the core variables, thereby making the estimation of the mediating pathways more rigorous. The findings are intended to inform the development of more precise and anticipatory psychological interventions based on empirical evidence. The corresponding theoretical model is depicted in Figure 1.

**FIGURE 1:**
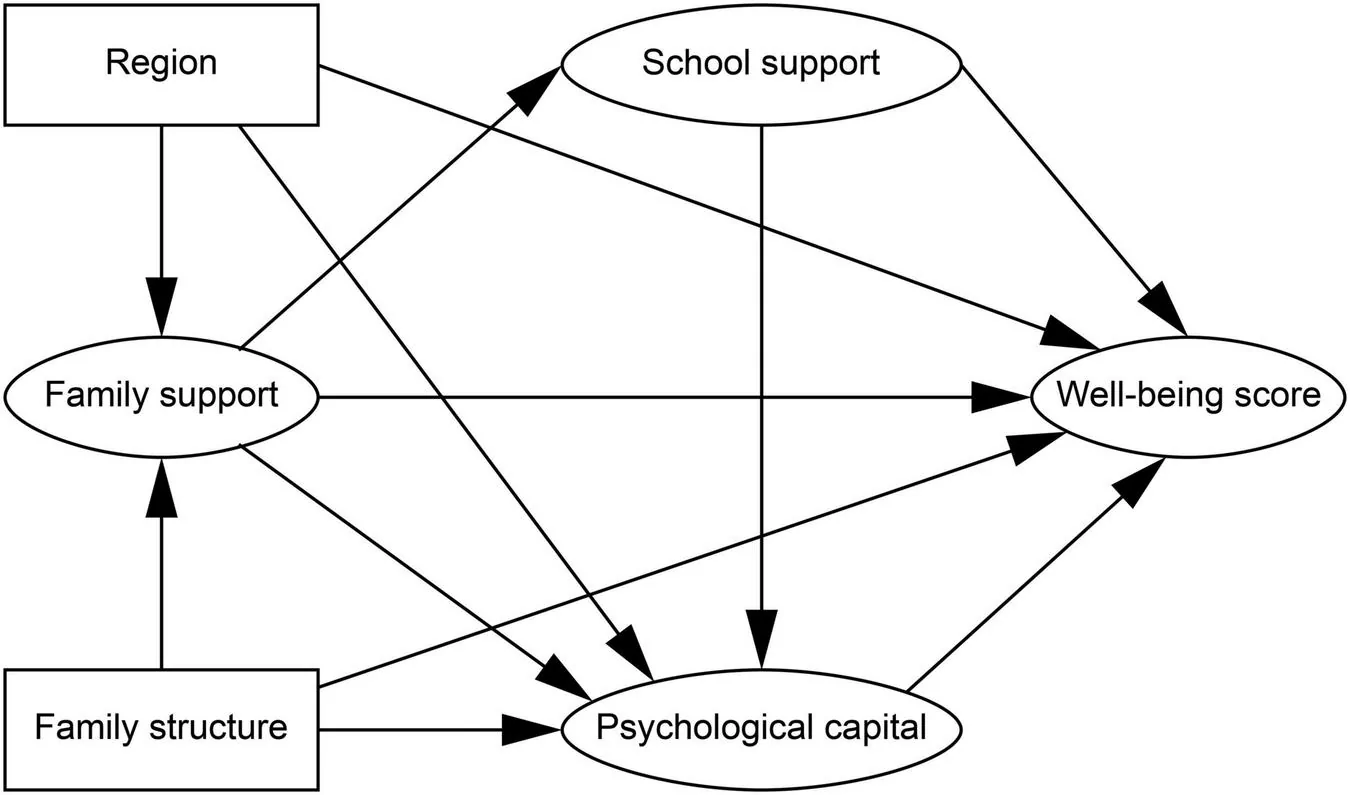
Theoretical model diagram.

## 2 Materials and methods

### 2.1 Measures

This study employed a self-administered questionnaire to conduct a cross-sectional survey. The first part of the questionnaire was used to collect demographic information, including university, grade, major, program level, gender, place of origin, family structure, father’s occupation, and mother’s occupation. The core research variables were measured using scales that had been validated for reliability and validity, as specified below:

### 2.1.1 Well-being: mental health continuum-short form (MHC-SF)

The Mental Health Continuum-Short Form (MHC-SF) was adopted to assess college students’ well-being. Originally developed by Keyes in 2002, this study utilized the Chinese version of the MHC-SF revised by Yin and He (2012); RRID:SCR_015805) to measure well-being among college students. Developed based on the positive mental health theory, the scale consists of 14 items covering three dimensions: emotional well-being, social well-being, and psychological well-being. A 5-point Likert scoring system was employed, on which participants rated the frequency of corresponding feelings experienced over the past month, ranging from 1 = “never” to 5 = “every day.” The total score ranges from 14 to 70, with higher total scores indicating greater well-being in college students. Satisfactory reliability and validity of this scale have been verified among Chinese college student samples (Xiao et al., 2021). In the present study, the Cronbach’s α coefficient of the scale was 0.958 and the KMO value was 0.963, indicating good reliability and construct validity.

### 2.1.2 Family support: family APGAR index

Family support was measured using the Family APGAR Index developed by Smilkstein et al. (1982); RRID:SCR_006567). Originally designed to assess family members’ satisfaction with family functioning, the scale covers five dimensions: Adaptation, Partnership, Growth, Affection, and Resolve. The Chinese version of the Family APGAR Questionnaire introduced by Lü et al. (1999) was adopted in the present study. This Chinese version has been applied and validated in multiple studies involving Chinese college students and clinical populations (Lü and Gu, 1995; He et al., 2024). The scale consists of 5 items, with each dimension corresponding to one item, rated on a 3-point Likert scale (1 = rarely, 2 = sometimes, 3 = often). Total scores range from 5 to 15, with higher scores indicating stronger perceived family support. It should be noted that although the Family APGAR Index was originally developed as a tool for assessing family functioning, its five dimensions operationally reflect the quality of supportive resources available to family members when facing difficulties (e.g., “I am satisfied with the help I receive from my family when I have problems” reflects instrumental support; “I am satisfied with the way my family expresses concern and affection toward my emotions” reflects emotional support). Previous studies have used this scale as a valid measure of perceived family support among college students (Arias-De la Torre et al., 2019). In this study, the Cronbach’s α coefficient of the scale was 0.935, and the KMO test value was 0.895, indicating good reliability and validity.

### 2.1.3 School support scale

The measurement of school support employed a self-developed School Support Scale. The scale’s development referenced Epstein’s (2018) theoretical framework of school–family partnership and social support (Fiore, 2001), and was adaptively adjusted to the Chinese higher education context. After literature analysis, expert review, and pilot testing, four items were finally retained, mainly reflecting core content such as students receiving help, accepting teachers’ ways of discussion, teachers providing support, and teachers showing care and concern. The scale uses a 3-point Likert scale (1 = rarely, 2 = sometimes, 3 = often), with total scores ranging from 4 to 12; higher scores indicate higher levels of perceived school support. This scale was developed specifically for the Chinese university context and corresponds to the actual teacher–student interaction scenarios in domestic colleges. In the sample of this study, the KMO value of the scale was 0.825, and Bartlett’s test of sphericity reached a significant level (χ^2^ = 27465.96, df = 6, *P* < 0.001). Exploratory factor analysis showed that the variance explained by the single factor was 77.38%, indicating that the construct validity met psychometric requirements. The overall Cronbach’s α coefficient of the scale was 0.871, demonstrating good internal consistency.

### 2.1.4 Psychological capital scale

The measurement of psychological capital was based on the four-dimensional theoretical framework of positive psychological capital proposed by Luthans et al. (2007). The operationalization was implemented using a simplified approach that selected and matched items from the Positive Affect subscale of the Chinese version of the Positive and Negative Affect Schedule (PANAS). The theoretical rationale for this measurement approach rests on the fact that psychological capital, as a state-like positive psychological resource (Luthans and Youssef-Morgan, 2017), has core components that conceptually overlap with state positive affect at the experiential level. Fredrickson’s (2004) broaden-and-build theory points out that positive emotional experiences can broaden an individual’s momentary thought-action repertoires and build enduring personal psychological resources (including core elements of psychological capital such as resilience and optimism), thus providing theoretical support for using positive affect items to represent psychological capital. This simplified operationalization drew upon the paradigm of mapping state-positive-affect items onto core components of psychological capital as used in previous studies (Avey et al., 2010), and was adapted for item matching according to the psychological characteristics of Chinese college students.

The Chinese version of the PANAS was originally developed by Watson et al. (1988) and revised by Huang et al. (2003). It has been widely used and validated in Chinese populations, with stable reliability and validity (Cronbach’s α coefficients for positive and negative affect were 0.85 and 0.83 respectively, and 0.82 for the full scale; test-retest reliability was 0.47). The scale comprises 8 items covering four core dimensions: Resilience (3 items: “determined,” “attentive,” “active”); Optimism (2 items: “enthusiastic,” “vigorous”); Hope (2 items: “excited,” “proud”); and Self-efficacy (1 item: “inspired”). All items are rated on a 5-point Likert scale (1 = very slightly or not at all, 5 = extremely). The total psychological capital score is the sum of all item scores; higher scores indicate higher levels of individual psychological capital.

In the sample of this study, the Cronbach’s α coefficient for the total psychological capital scale was 0.892. The Cronbach’s α coefficients for the subscales were: Resilience 0.895, Optimism 0.870, Hope 0.825; Self-efficacy consisted of a single item and thus internal consistency was not computed. To examine the construct validity of this operational measurement scheme, confirmatory factor analysis (CFA) was conducted to evaluate the fit of the four-factor model. The model was estimated using Maximum Likelihood Estimation, and the following fit indices are reported: χ^2^/df = 55.486, Comparative Fit Index (CFI) = 0.988, Tucker-Lewis Index (TLI) = 0.978, Root Mean Square Error of Approximation (RMSEA) = 0.071 (90% CI [0.067, 0.075]), and Standardized Root Mean Square Residual (SRMR) = 0.014. According to the recommendations of Hu and Bentler (1999) and Browne et al. (1993), good model fit is indicated by CFI ≥ 0.95, TLI ≥ 0.95, RMSEA ≤ 0.06 (acceptable range ≤ 0.08), SRMR ≤ 0.08, and χ^2^/df < 3. In summary, except for the χ^2^/df value, which was influenced by the large sample size, all other core fit indices reached the established criteria, indicating that the four-factor CFA model fit well and supported the construct validity of this operationalization scheme. It should be noted that this measurement is not a dedicated psychological capital scale, but rather a theoretically driven operationalization using positive affect items from the PANAS; its content validity may be weaker than that of dedicated psychological capital instruments such as the PCQ-24. Therefore, the interpretation of the psychological capital results in this study is made with caution.

### 2.2 Data source

This study is a large-scale cross-sectional survey. First, to ensure the representativeness of the sample for the college student population in Shandong Province, the formula for sample size estimation based on an infinite population proportion was used to calculate the initial sample size. The calculation formula is as follows:

The significance level was set at α = 0.05, with the corresponding standard normal Z-value of 1.96; the expected population proportion π was conservatively taken as 0.5; and the allowable error e was 0.01. Substituting these values into the formula yielded a baseline sample size of n_0_ = 9,604. Given that this study adopted a multi-stage cluster sampling design, which produces larger sampling errors than simple random sampling, a design effect (DEFF = 1.5) was introduced for adjustment. Additionally, a 10% invalid response rate was presupposed. Based on these adjustments, the theoretical minimum sample size was calculated as approximately 9,604 × 1.5/0.9 ≈ 16,006 cases. However, in actual practice, constrained by the objective cluster size of administrative classes as the smallest sampling units in cluster sampling and the feasibility of field survey implementation, the final target sample size for this study was set at no less than 11,500 cases. For the core analyses of this study–multiple linear regression, Bootstrap mediation effect, and structural equation modelling–the final effective sample size was 10,726 cases. This effective sample size not only far exceeds the general rule of thumb for structural equation modelling that the sample size should be 5 to 10 times the number of observed variables, but also meets the large-sample power criteria for mediation effect testing, ensuring sufficient statistical power.

Regarding sampling implementation, the survey was conducted in Shandong Province from October to December 2024. Based on the Shandong Statistical Yearbook, the 16 prefecture-level cities in the province were first stratified into three levels (high, medium, and low) according to the density of higher education institutions, and were simultaneously cross-stratified by institution type (undergraduate/vocational college). The specific sampling was carried out in three stages:

Stage 1 (Selecting institutions): According to the proportional distribution of geographic strata and institution-type strata, 8 institutions were randomly selected. Specifically, 3 undergraduate institutions were selected from high-density areas; 2 undergraduate and 2 vocational institutions from medium-density areas; and 1 undergraduate institution from low-density areas. In total, 6 undergraduate institutions and 2 vocational institutions were included.

Stage 2 (Selecting departments/schools): Within each selected institution, a cluster sampling method was used to select several departments/schools, covering all undergraduate students from Year 1 to Year 5.

Stage 3 (Selecting classes): Systematic sampling was applied to all classes within the above-selected departments/schools, ultimately identifying 11,500 students as survey respondents, and questionnaire distribution and collection were completed.

The inclusion and exclusion criteria are shown in Figure 2. The final effective sample size was 10,726 responses, yielding an effective response rate of 93.3%.

**FIGURE 2:**
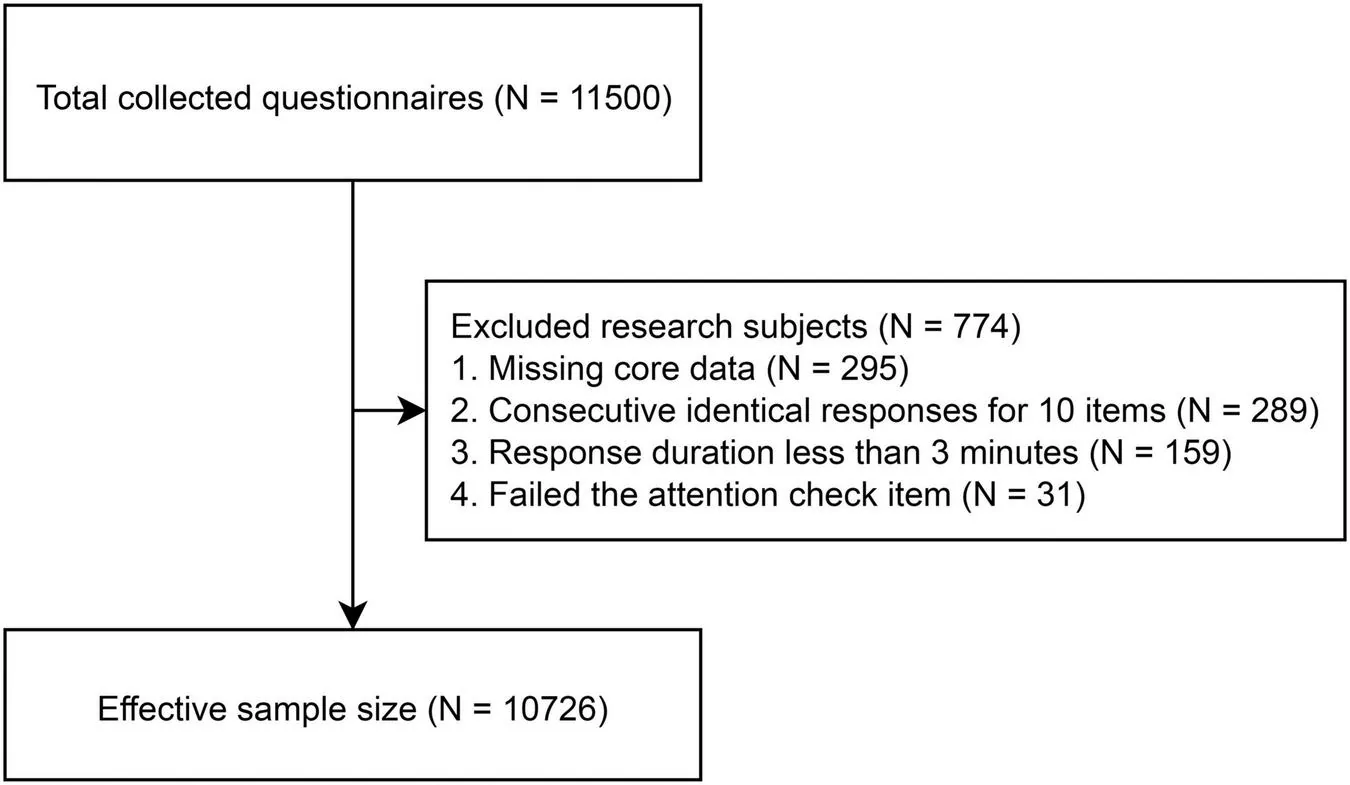
Flowchart of sample selection.

This study was approved by the Ethics Committee of Shandong Second Medical University (Approval No.: 2025YX69, Date of Approval: September 12, 2024). The entire study was conducted in accordance with the Declaration of Helsinki and relevant local laws, regulations, and institutional requirements. All participants provided written informed consent prior to data collection and voluntarily participated in this study.

### 2.3 Statistical analysis

Statistical analyses were performed using SPSS (version 26.0, RRID:SCR_002865). Sample characteristics were described using frequencies (n) and percentages (%). For variables with a small number of missing values on parental occupation, listwise deletion was applied, and all analyses were conducted based on valid cases for each variable. The proportion of missing values was less than 8%, which is unlikely to introduce systematic bias into the results. The difference in sample sizes stemmed from structural missingness in the parental occupation variables: among the total sample of 10,726 participants, 815 came from single-parent families, for whom occupation information for one parent was naturally absent. Therefore, descriptive statistics and between-group comparisons of parental occupation were performed only among the 9,911 participants from two-parent families. Differences in well-being scores across demographic characteristics were examined using independent-samples *t-*tests and one-way analysis of variance (ANOVA). Multiple linear regression was employed to identify potential determinants of well-being scores.

Given the multi-stage stratified cluster sampling design, in which the sample was nested within three levels (school, department, and class), the intraclass correlation coefficient (ICC) at the class level was calculated to assess the potential influence of clustering effects on the analytical results. The results showed that the ICC for well-being scores within classes was approximately 0.01, indicating that about 1.00% of the total variance could be explained by between-class differences, suggesting relatively small clustering effects. Furthermore, sensitivity analyses were performed using cluster-robust standard errors with class as the clustering variable. The results were largely consistent with the main analyses (family support: *B* = 2.404, *P* < 0.001; school support: *B* = 0.566, *P* = 0.004; psychological capital: *B* = 0.986, *P* < 0.001), indicating that clustering effects had limited impact on the primary conclusions of this study; detailed results are presented in Supplementary Material 1.

SEM was conducted using AMOS (version 28.0, RRID:SCR_016253) to explore the relationships among the influencing factors of college students’ well-being. The Bootstrap method (with 5,000 resamples) was used to test the mediating effects of psychological capital and school support in the association between family support and well-being. The significance level for all tests was set at α = 0.05.

## 3 Results

### 3.1 Comparative analysis of well-being among college students

A univariate analysis was conducted with the well-being score as the dependent variable and the following factors as independent variables: grade level, major, academic program type, gender, place of origin, family structure, mother’s work unit, and father’s work unit. The results revealed statistically significant differences in well-being scores across grade level, major, academic program type, family structure, and parental work units (*P* < 0.05). These results are presented in Table 1.

**TABLE 1:** Analysis of differences in well-being of college students.

Variable	Group	Frequency	Percentage	Well-being (M ± SD)	Test statistic (*df*)	*P*	Effect size
Academic year	Year 1	6481	60.40%	59.09 ± 12.01	*F*(4,10721) = 15.098	<0.001	η^2^ = 0.005
	Year 2	2718	25.30%	59.95 ± 12.37			
	Year 3	710	6.60%	61.75 ± 10.55			
	Year 4	680	6.30%	61.59 ± 10.63			
	Year 5	137	1.30%	61.84 ± 11.33			
Major	Medical sciences	1840	17.20%	60.48 ± 12.67	*F*(4,10721) = 6.386	<0.001	η^2^ = 0.002
	Natural sciences	905	8.40%	58.28 ± 11.02			
	Engineering	2634	24.60%	59.99 ± 12.11			
	Humanities and social sciences	2195	20.50%	59.23 ± 11.61			
	Other	3152	29.40%	59.67 ± 11.94			
Program type Gender	Associate degree	5944	55.40%	59.10 ± 12.70	*t*(10724) = −5.571 *t*(10724) = 0.262	<0.001 0.793	Cohen’s d = 0.108 Cohen’s d = 0.005
	Bachelor’s degree	4782	44.60%	60.39 ± 10.93			
	Women	4744	44.20%	59.71 ± 11.25			
	Men	5982	55.80%	59.65 ± 12.49			
Place of origin	Urban	2775	25.90%	60.04 ± 11.82	*t*(10724) = 1.862	0.063	Cohen’s d = 0.041
	Rural	7951	74.10%	59.55 ± 12.00			
Family structure	Dual-parent family	9911	92.40%	59.84 ± 11.85	*t*(10724) = 4.732	<0.001	Cohen’s d = 0.172
	Single-parent family	815	7.60%	57.78 ± 13.00			
Mother’s occupation	Unemployed	902	8.40%	58.27 ± 13.24	*F*(5,9905) = 11.163	<0.001	η^2^ = 0.006
	Civil servant	116	1.10%	62.5 ± 10.90			
	Public institution	1053	9.80%	59.66 ± 11.81			
	Enterprise	1156	10.80%	60.12 ± 11.70			
	Farmer	3462	32.30%	59.33 ± 11.97			
	Self-employed	3222	30%	60.67 ± 11.34			
Father’s occupation	Unemployed	283	2.60%	56.94 ± 15.17	*F*(5,9905) = 10.276	<0.001	η^2^ = 0.006
	Civil servant	201	1.90%	60.89 ± 11.45			
	Public institution	1178	11%	60.26 ± 11.49			
	Enterprise	1500	14%	59.84 ± 11.69			
	Farmer	3374	31.50%	59.21 ± 12.14			
	Self-employed	3375	31.50%	60.49 ± 11.40			

The effective sample size for mother’s occupation and father’s occupation was 9,911 (only for students from two-parent families), whereas the effective sample size for the other variables was 10,726. The significance level was set at *P* < 0.05.

### 3.2 Linear regression analysis of factors influencing college students’ well-being scores

Taking well-being as the dependent variable, a multiple linear regression was conducted with family support, school support, and psychological capital as independent variables. The results of Model 3 indicated that grade, major, gender, place of origin, family support, teacher support, and psychological capital were all significantly correlated with well-being. Among these, psychological capital, family support, and school support were positively associated with well-being. Notably, the standardized regression coefficient (β) of psychological capital (β = 0.567) was the largest among all predictors in the model, followed by family support (β = 0.185) and school support (β = 0.110), suggesting that psychological capital had a relatively stronger association with well-being. The results are presented in Table 2.

**TABLE 2:** Multiple linear regression analysis of well-being scores of college students^a^.

Variable	B	S.E.	β	95%CI	*P*
Model 1 (demographic control variables)					
Academic year	0.835	0.131	0.066	[0.579, 1.092]	< 0.001
Major (reference group: other)					
Medical sciences	−1.107	0.446	−0.035	[−1.982, −0.232]	0.013
Natural sciences	−1.883	0.462	−0.044	[−2.788, −0.977]	< 0.001
Engineering	−0.195	0.328	−0.007	[−0.839, 0.449]	0.553
Humanities and social sciences	−0.933	0.354	−0.031	[−1.627, −0.238]	0.008
Program type (bachelor’s degree = 1)	1.254	0.290	0.052	[0.686, 1.822]	< 0.001
Gender (men = 1)	0.111	0.251	0.005	[−0.382, 0.603]	0.660
Place of origin (rural = 1)	−0.249	0.267	−0.009	[−0.772, 0.273]	0.350
Family structure (single-parent family = 1)	−2.090	0.434	−0.046	[−2.941, −1.239]	< 0.001
Adjusted R^2^	0.011				
Model 2 (demographic control variables+family support)					
Academic year	0.553	0.113	0.044	[0.333, 0.774]	< 0.001
Major (reference group: other)					
Medical sciences	−1.363	0.384	−0.043	[−2.116, −0.611]	< 0.001
Natural sciences	−1.680	0.397	−0.039	[−2.458, −0.901]	< 0.001
Engineering	−0.429	0.282	−0.015	[−0.983, 0.125]	0.129
Humanities and social sciences	−1.111	0.305	−0.038	[−1.709, −0.514]	< 0.001
Program type (bachelor’s degree = 1)	0.859	0.249	0.036	[0.370, 1.348]	0.001
Gender (men = 1)	−0.039	0.216	−0.002	[−0.463, 0.385]	0.857
Place of origin (rural = 1)	0.306	0.230	0.011	[−0.144, 0.756]	0.183
Family structure (single-parent family = 1)	−0.472	0.374	−0.010	[−1.205, 0.262]	0.208
Family support	2.396	0.039	0.511	[2.320, 2.473]	< 0.001
Adjusted R^2^	0.268				
Model 3 (demographic control variables+family support+school support+psychological capital)					
Academic year	0.345	0.088	0.027	[0.172, 0.519]	< 0.001
Major (reference group: other)					
Medical sciences	−1.022	0.302	−0.032	[−1.614, −0.431]	0.001
Natural sciences	−1.051	0.312	−0.024	[−1.663, −0.439]	0.001
Engineering	−0.321	0.222	−0.012	[−0.756, 0.114]	0.148
Humanities and social sciences	−0.521	0.239	−0.018	[−0.990, −0.052]	0.030
Program type (bachelor’s degree = 1)	0.014	0.196	0.001	[−0.370, 0.399]	0.941
Gender (men = 1)	−1.693	0.171	−0.070	[−2.028, −1.358]	< 0.001
Place of origin (rural = 1)	0.584	0.180	0.021	[0.230, 0.937]	0.001
Family structure (single-parent family = 1)	−0.400	0.294	−0.009	[−0.976, 0.177]	0.174
Family support	0.868	0.039	0.185	[0.791, 0.944]	< 0.001
School support	0.572	0.044	0.110	[0.487, 0.658]	< 0.001
Psychological capital	0.983	0.014	0.567	[0.957, 1.010]	< 0.001
Adjusted R^2^	0.548				

^a^Dependent variable: well-being. S.E. refers to standard error; CI denotes confidence interval.

### 3.3 Construction and validation of the structural equation model for college students’ well-being

#### 3.3.1 Structural equation modeling analysis

This study, grounded in research hypotheses and theoretical framework, took college students’ well-being as the dependent variable, family support as the core independent variable, school support and psychological capital as serial mediators, and place of origin and family structure as exogenous control variables. A structural equation model (SEM) was constructed using AMOS 28.0, with model parameters estimated and fit validated via maximum likelihood estimation, to systematically examine the interrelationships among the variables.

#### 3.3.2 Specification of observed indicators for latent variables

In terms of model indicator specification, school support was treated as a unidimensional latent variable, with its four corresponding measurement items directly used as observed indicators. Psychological capital was treated as a multidimensional latent variable; following the standardized practice of SEM, the parceling approach was adopted, using the dimension means of resilience, optimism, hope, and self-efficacy as observed indicators. The rationale and advantages of this approach have been widely validated in the literature, with the key justifications as follows: (1) parceling effectively reduces random measurement error inherent in single items, thereby enhancing the reliability of observed indicators and overall model stability; (2) given the sufficient sample size in this study, dimension-aggregated parceling simplifies the model structure, reduces the number of parameters to be estimated, and avoids overfitting, while fully preserving the theoretical connotations of the four-dimensional structure of psychological capital, in line with mainstream modeling practices (Little et al., 2002). It should be noted that the self-efficacy dimension of psychological capital contained only one measurement item; therefore, that item was directly included as the corresponding observed indicator in the model.

#### 3.3.3 Rationale for including exogenous variables

In the SEM, only variables with direct theoretical relevance and statistically significant associations with the core path (family support → well-being) were retained as exogenous variables to ensure model identifiability and avoid unnecessary complexity. Specifically, place of origin (marginally significance, *P* = 0.063) and family structure (*P* < 0.001) were included as exogenous variables because they are directly related to the accessibility of family support. Although parental occupation was significant in univariate analyses, it was not entered into the SEM because it conceptually overlaps with the family support scale (both reflect family resource dimensions), which could introduce multicollinearity. Grade, major, and educational level mainly reflect academic-stage characteristics and have weak theoretical links to the core path, so they were not included. Gender was not significant in univariate analysis (*P* = 0.793) and was also excluded from the SEM.

#### 3.3.4 Initial model construction and path deletion

Model construction and refinement strictly followed the principles of theoretical support, data fit, and model parsimony. The specific revision process was as follows: First, an initial SEM was built based on the initial theoretical hypotheses, presetting direct paths from family support to college students’ well-being, school support, and psychological capital, while also specifying the mediating effects of school support and psychological capital and the control effects of exogenous variables. The parameter estimation results of the initial model showed that the regression coefficients for the paths from family structure to psychological capital (*P* = 0.085) and from family structure to well-being (*P* = 0.329) did not reach statistical significance. To adhere to the principle of model parsimony and eliminate interference from invalid paths, these two non-significant paths were deleted.

#### 3.3.5 Residual covariance modification based on modification indices

To control for measurement error covariance issues arising from common method bias and further optimize model fit, modification indices (MI) were used for fine-tuning. The modification followed two strict criteria: (1) MI > 15 for the residual covariance, and (2) the covariance-linked measurement items belonged to the same latent variable and the same core dimension, with sufficient theoretical justification. The specific modifications were as follows: In the family support dimension, covariances between residual terms of items measuring intimacy, emotional support, and cooperative support were added (e1–e2, e2–e4), as these items all focus on daily emotional interactions within the family and share common-source specific variance. In the school support dimension, covariances between residual terms of items measuring teacher–student communication, encouragement, and care were added (e11–e12, e11–e13, e12–e13), consistent with the common-source measurement characteristics of interpersonal teacher–student interactions. In the psychological capital dimension, covariances between residual terms of items measuring optimism, self-efficacy, and resilience were added (e6–e7, e7–e9), as these three dimensions all belong to individual positive psychological resources and share a higher-order psychological factor of “positive cognition–stress coping.” In the well-being dimension, covariance between residual terms of items measuring emotional well-being and psychological well-being was added (e14–e16), as both are influenced by unmodeled latent factors such as stable personality traits and environmental perceptions. These modifications only optimized the covariances of item-specific variances without altering the core theoretical paths among variables, effectively avoiding contamination of latent variable relationship estimates by common method bias and ensuring the accuracy of parameter estimation.

#### 3.3.6 Final model fit results

After path deletion and residual covariance modification, the final structural equation model was obtained (see Figure 3). Model fit indices showed that the initial model had CFI = 0.981 and RMSEA = 0.044; after modification, the final model fit improved to CFI = 0.990 and RMSEA = 0.034, indicating a marked enhancement. The fit indices for the initial model are presented in Supplementary Material 2, and those for the final model are shown in Table 3.

**FIGURE 3:**
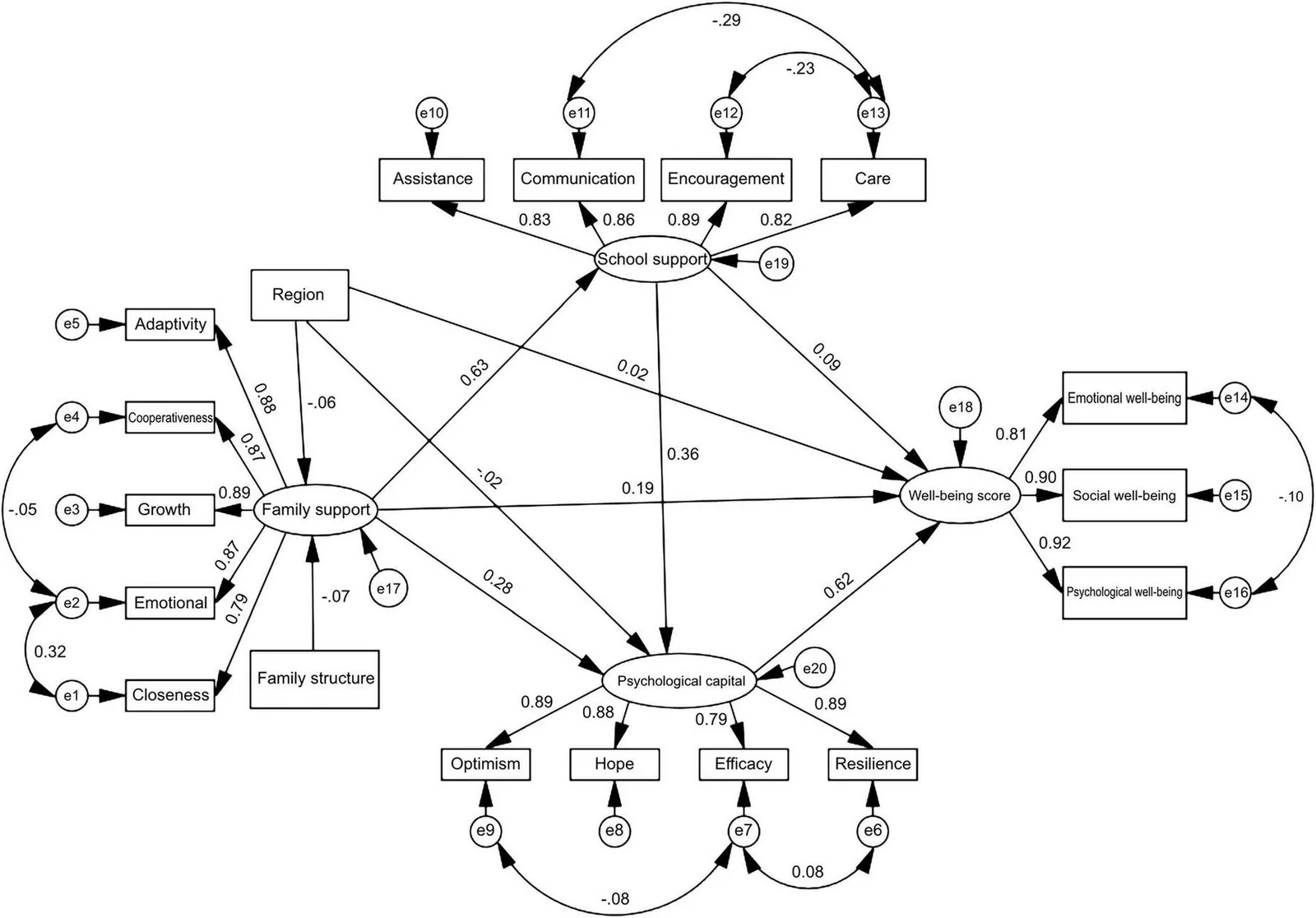
Structural equation model (SEM) of factors influencing college students’ mental health.

**TABLE 3:** Goodness-of-Fit indices for the structural equation model.

Indicator	χ^2^	df	χ^2^/df	GFI	AGFI	NFI	RFI	IFI	TFI	CFI	RMSEA	RMSEA90%CI
Recommended criteria	–	–	< 5 (good)	> 0.9	>0.9	> 0.9	>0.9	> 0.9	>0.9	> 0.9	< 0.05	–
Obtained values	1573.752	120	13.115	0.984	0.977	0.989	0.986	0.99	0.987	0.990	0.034	0.032–0.035

Due to the large sample size (*N* = 10,726), the χ^2^ test tends to be significant; thus, we mainly relied on CFI, TLI, RMSEA, and SRMR to evaluate model fit. Overall, these fit indices suggest that the final model demonstrates good fit to the data.

### 3.4 Path analysis of structural equation model for college students’ well-being

Results of the structural equation model indicated that all paths were statistically significant (*P* < 0.05). Family support was positively associated with well-being (path coefficient = 0.186), which supported Hypothesis H1. In terms of path coefficients, family support was positively correlated with psychological capital as well as well-being scores. This suggests that the positive association between family support and well-being can function through psychological capital as a mediating variable. Among all paths examined in the model, the mediating effect corresponding to “Family Support → Psychological Capital → Well-Being” exhibited the largest effect size.

Path analysis of exogenous variables revealed that family structure was negatively correlated with family support (β = −0.071, *P* < 0.001), indicating that college students from single-parent families perceived significantly lower family support than those from non-single-parent families. Place of origin was negatively correlated with family support (β = −0.056, *P* < 0.001) and psychological capital (β = −0.018, *P* = 0.029), but positively correlated with well-being (β = 0.024, *P* < 0.001). Taken together, Hypothesis H5 was only partially supported. Specifically, students from single-parent families reported significantly lower perceived family support, and students with rural origins had significantly lower levels of family support and psychological capital, consistent with the hypothesized directions. Nevertheless, rural-origin students demonstrated significantly higher well-being than urban-origin students, contrary to the predicted direction of the hypothesis. Direct paths from family structure to psychological capital and well-being were non-significant. The results are presented in Table 4.

**TABLE 4:** Path coefficients of the factors influencing college students’ well-being based on SEM.

Path	Standardized path coefficient	Unstandardized path coefficient	S.E.	*p*
Family structure → family support	−0.071	−0.109	0.015	< 0.001
Place of origin → family support	−0.056	−0.052	0.009	< 0.001
Family support → school support	0.625	0.818	0.014	< 0.001
School support → psychological capital	0.357	1.604	0.053	< 0.001
Place of origin → psychological capital	−0.018	−0.101	0.046	0.029
Family support → psychological capital	0.281	1.654	0.07	< 0.001
Place of origin → well-being	0.024	0.119	0.033	< 0.001
Family support → well-being	0.186	0.983	0.052	< 0.001
Psychological capital → well-being	0.620	0.558	0.009	< 0.001
School support → well-being	0.087	0.351	0.04	< 0.001

S.E., standard error.

### 3.5 Mediating effect analysis of variables related to college students’ well-being

To further explore the association mechanism among variables related to college students’ well-being, the Bootstrap method (5000 resamples) was adopted in this study to test the mediating effects. The results indicated that school support and psychological capital exerted independent mediating effects respectively. Meanwhile, the two variables formed a serial mediating path between family support and well-being, and none of the bias-corrected Bootstrap 95% confidence intervals contained zero. The combined effect values of these three paths accounted for 66.4% of the total effect. These findings confirmed that the respective mediating effects of school support and psychological capital, as well as their serial mediating path, were significant in the relationship between family support and students’ well-being. Accordingly, Hypotheses H2, H3, and H4 were verified. Detailed results are shown in Table 5.

**TABLE 5:** Results of the mediation effect test.

Path	Estimate	95% CI	*p*	Proportion of total effect (%)
Direct effect	0.982	0.829∼1.062	–	33.6
Indirect effect	1.942	1.734∼1.949	–	66.4
Total effect	2.924	2.648∼2.928	–	100.0
Family support → school support → well-being	0.287	0.213∼0.361	< 0.001	9.8
Family support → school support → psychological capital → well-being	0.732	0.667∼0.799	< 0.001	25.0
Family support → psychological capital → well-being	0.923	0.832∼1.024	< 0.001	31.6

CI, confidence interval.

## 4 Discussion

### 4.1 Direct effect of family support on college students’ well-being

The present study’s findings demonstrate that family support was significantly positively correlated with college students’ well-being. aligning with the existing body of literature and confirming the established role of family support (Schnettler et al., 2015). First, the family serves as the primary milieu for individual development, functioning as the initial context in which emotional experiences are accumulated and interpersonal interactions are learned. For students, the family provides emotional sustenance and reliance, enabling them to seek solace and encouragement from such relationships when confronting academic and life stressors. This support system effectively mitigates the emergence of negative emotions such as anxiety and depression (Chen et al., 2023). Furthermore, this emotional support fosters the development of a secure attachment style, which predisposes individuals to interpret negative events through a more positive and constructive lens. This adaptive cognitive pattern constitutes a fundamental basis for the formation of sustained well-being. Moreover, family support extends to tangible financial assistance and informed decision-making counselling. For university students, stable economic provision and experiential guidance from parents effectively mitigate future-oriented uncertainties, thereby alleviating anxiety and enhancing well-being (Armstrong and Hamilton, 2013). Family support is also typically accompanied by constructive family communication patterns and a positive family atmosphere. This nurturing environment fosters the development of psychological well-being and sound value systems in university students, thereby equipping them with greater resilience to cope with life’s adversities and challenges.

### 4.2 Mediating role of school support between family support and well-being

The results indicate that school support plays a significant mediating role in the relationship between family support and college students ‘well-being. High levels of family support facilitate positive home–school communication, enabling schools to better identify students’ needs and optimize the allocation of on-campus resources, thereby helping students receive stronger support from teachers and peers and ultimately enhancing their well-being (Siu et al., 2014; Epstein, 2018). When students encounter difficulties on campus, support from teachers, peers, and fellow students can help alleviate psychological stress and mitigate negative emotions; it serves as an important external factor in safeguarding college students’ well-being (Yan et al., 2011). However, the contribution ratio of this intermediary pathway is relatively lower, which is closely related to the developmental characteristics of college students during early adulthood (Arnett, 2000). Upon entering university, individuals’ demand for independence increases, while parents’ direct involvement in campus life declines significantly, thereby weakening the transmission of family support to school-based support. Furthermore, college students perceive school support as relying more heavily on their own social networks and proactive seeking of assistance, meaning that individual agency dilutes the influence exerted at the family level (Sasser et al., 2023). As a result, this independent mediating effect is relatively weak. Furthermore, the home-school collaboration model for university students differs from that employed in primary and secondary education settings. While parents in these earlier stages are deeply involved in school management and daily teaching activities, the home-school collaboration among university students is centered on information sharing, joint risk prevention, and collaborative development (Tian et al., 2022). Students with higher levels of family support tend to have their parents maintain long-term communication with counselors or mentors, enabling them to stay informed about the students’ academic and psychological well-being in a timely manner (Hill and Taylor, 2004). The school leverages a home–school collaborative early-warning and support system to provide targeted assistance. This two-way information exchange enables the precise allocation of resources and has become an important practical mechanism for integrating family support, school support, and students’ well-being. Hence, school support plays a critical role in the relationship between family support and well-being scores.

### 4.3 Mediating role of psychological capital between family support and well-being

The results demonstrate a statistically significant mediating effect of psychological capital in the relationship between family support and well-being scores. It is noteworthy that the indirect effect accounts for a relatively large proportion of the total effect. This finding is consistent with the report by Avey et al. (2011), suggesting that psychological capital may serve as an important psychological resource mediating the relationship between family support and well-being. As a positive psychological resource, psychological capital may function by transforming external family support into internalized psychological assets, thereby being associated to some extent with stress reduction and higher well-being; individuals with high psychological capital exhibit four key characteristics: a tendency to view challenges as opportunities for growth, the establishment and pursuit of meaningful goals, the ability to bounce back from setbacks through resilience, and the capacity to draw valuable lessons from adversity (Luthans and Broad, 2022).

Therefore, psychological capital may not merely serve as a passive barrier against negative influences; it can also represent the psychological resources through which individuals actively adapt to their environment and pursue well-being. As such, even though external support from family and school holds significant value, college students ‘sense of well-being remains closely linked to their psychological capital – reflecting an individual’s personal capacity to transform these supportive resources into positive cognitions and proactive behaviors. These findings suggest a paradigm shift in intervention strategies: rather than focusing predominantly on providing external support, we should partially redirect efforts toward helping students develop the capacity to internalize and transform existing support, thereby cultivating their positive psychological resources. This approach provides direction for achieving more fundamental and sustainable mental health promotion.

### 4.4 Chain mediating role of school support and psychological capital between family support and well-being

The study findings indicate that school support and psychological capital serve as a chain-mediated mechanism between family support and well-being. As a crucial environment for college students’ academic and personal development, the university may be associated with students’ level of psychological capital through its provision of educational resources, social opportunities, and structured activities, thereby establishing a chain-mediated relationship with well-being. Seligman (2006) demonstrated that teachers can foster students’ psychological capital by guiding them toward adaptive attribution styles—for instance, attributing successes to internal factors such as effort and ability, while interpreting failures as resulting from temporary or situational causes rather than inherent lack of competence. This helps sustain an optimistic disposition and improve overall psychological capital levels. The chain-of-cause pathway identified in this study sheds light on the potential transmission mechanisms linking external support resources, individual psychological capital, and well-being, providing empirical insights into the dynamic process through which university students’ well-being is shaped.

The research findings suggest that interventions aimed at enhancing college students’ well-being should move beyond a single environmental perspective and instead establish a multi-level collaborative framework—where the family serves as the foundational starting point, schools as the extended context, and psychological capital acts as the key link—thereby fostering synergistic improvement across these interconnected systems.

### 4.5 Family structure and geographic origin: structural disparities in well-being

This study incorporates place of origin and family structure as exogenous variables into the model, verifying the systematic impact of early structural factors on college students’ subjective well-being. This finding enriches existing research on the heterogeneity of family backgrounds. The influence of family structure on well-being is primarily indirect, mediated by reduced family support. Single- parent families often face challenges such as diminished family functioning and insufficient emotional support. The absence of one parent limits the companionship, communication, and financial support available to children, thereby lowering their perceived family support and subsequently affecting well-being through chain pathways involving school support and psychological capital. This aligns with Ren et al. (2025), who found that family disruption negatively affects long-term mental health by reducing the availability of social support. The influence of place of origin operates through multiple pathways. On the one hand, rural students report significantly lower levels of family support and psychological capital compared with their urban counterparts, which is closely related to disparities in economic conditions, educational philosophies, and social resources between urban and rural families. Rural families tend to prioritize material support while remaining relatively limited in emotional communication and psychological guidance, which hinders the accumulation of positive psychological capital (Zhang et al., 2017).

On the other hand, in response to the finding that H5 is not fully supported, this study posits that the core reason lies in the positive correlation between place of origin and well-being, which can be explained by the theory of relative deprivation and expectation differences. When rural students enter university, their living environment and access to resources have significantly improved compared to their previous circumstances; as a result, they experience a stronger sense of relative gain and consequently exhibit higher levels of well-being, thereby offsetting the negative impact of insufficient family support. The direct effect of family structure on well-being is not statistically significant, indicating that the influence of family structure operates primarily through the mediating variable of family support rather than acting directly on well-being. This aligns with the theoretical framework of the Family Stress Model, which states, the influence of family structure does not act directly on children’s well-being but is transmitted through mediating processes such as family support (Conger and Donnellan, 2007). In other words, the potential impact of single-parent families on their children’s well-being stems from the fact that being from a single-parent household may reduce the level of family support, rather than from the notion that being “single-parent” itself necessarily leads to a decline in well-being.

### 4.6 Limitations

This study has several limitations. First, regarding the measurement of psychological capital, this study uses the positive emotion items from the PANAS scale as a proxy measure of psychological capital. However, these PANAS items capture only transient, situational positive emotions and cannot fully encompass the cognitive-motivational core of psychological capital—such as goal-directed agency, cognitive resilience in adversity, and adaptive attributional styles—thus posing a risk of inadequate content validity. Although the confirmatory factor analysis indicated acceptable model fit, this operationalization can only be regarded as a theoretically driven, simplified proxy measure of psychological capital; its content validity cannot be equated with that of specialized psychological capital scales. Future research is encouraged to prioritize the use of well-validated, domain-specific psychological capital scales, such as the PCQ24. Second, important variables may have been omitted. Some significant factors affecting college students’ well-being may not have been included in the current survey. Future research should consider employing more comprehensive assessments. Third, this study adopted a cross-sectional design, with all variables measured at a single time point, which can only reveal statistical associations among variables and cannot establish strict temporal precedence or causality. The direction of the mediation pathways proposed in this study was based on existing theoretical frameworks, and the possibility of reverse causation cannot be ruled out. Future research should adopt longitudinal designs and use multi-wave data to verify the causal order and direction of the pathways. Finally, all data were derived from self-report questionnaires. Although procedural controls such as anonymous administration and reverse-scored items were implemented, common method bias cannot be entirely eliminated. Subsequent studies could incorporate multi-wave surveys or multi-source data cross-validation to further test the robustness of the findings.

## 5 Conclusion

This study develops a chain-mediated model to explore the relationship between family support and college students’ well-being. The results indicate that, despite the diverse family backgrounds of the students, family support is not only directly positively correlated with well-being but also statistically mediates this relationship through three distinct indirect pathways: the independent mediating role of school support, the independent mediating role of psychological capital, and an ordered mediating pathway described as “Family Support → School Support → Psychological Capital → Well-being.” Among these pathways, psychological capital serves as the primary mediating variable, which aligns with the hypothesized pathway that “external support must be transformed into positive internal psychological resources before it can be associated with well-being.” These findings clarify how family support influences well-being through multiple mediating mechanisms and further suggest that interventions aimed at enhancing college students’ well-being should prioritize the significant role of psychological capital.

## 6 Resource identification initiative

All Research Resource Identifiers (RRIDs) were retrieved and verified from the SciCrunch Resource Portal:

IBM SPSS Statistics for Windows (Version 26.0): RRID:SCR_002865

IBM SPSS Amos (Version 28.0): (RRID:SCR_016253)

### 6.1 Sampling framework and data sources

#### 6.1.1 Shandong statistical yearbook 2024

Publisher: Shandong Provincial Bureau of Statistics, China Statistics

Press RRID: Not available

Public Access Link: http://tjj.shandong.gov.cn/col/col14603/index.html

Use: Stratification of higher education institution density across 16 prefecture-level cities for multi-stage stratified cluster sampling.

#### 6.1.2 Original study dataset description: cross-sectional dataset of 10,726 full-time college students in Shandong Province (collected October–December 2024)

RRID: Not applicable (study-specific original dataset)

Access: Available from corresponding authors upon reasonable request, subject to institutional ethics committee oversight (see full Data Availability Statement)

### 6.2 Data collection and quality control tools

#### 6.2.1 Self-administered structured questionnaire

Developer: The study research team

RRID: Not applicable

Use: Collection of demographic characteristics, family support, school support, psychological capital and subjective well-being data (Supplementary Data 1.1).

## Data Availability

The raw data supporting the conclusions of this article will be made available by the authors, without undue reservation.
