# Supplementary material for: The association between family support and college students’ well-being: an analysis of mediating pathways
Source: Front Psychol. 2026 Sep 18;17:1860654. doi: 10.3389/fpsyg.2026.1860654 (PMC13630776; doi:10.3389/fpsyg.2026.1860654)
Supplement: Supplementary file 1 [file Data_Sheet_1.docx]

| **Supplementary Material S1: Cluster-robust standard errors, with clustering at the class level.** | | | | | |
| --- | --- | --- | --- | --- | --- |
| Variable | B | S.E. | *β* | 95%CI | *P* |
| Model 1 |  |  |  |  |  |
| Major(reference group: Other) |  |  |  |  |  |
| Medical Sciences | -0.544 | 0.599 | -0.035 | [-2.207,1.119] | 0.415 |
| Natural Sciences | -1.911 | 0.193 | -0.044 | [-2.448,-1.375] | 0.001 |
| Engineering | -0.184 | 0.192 | -0.007 | [-0.719,0.350] | 0.393 |
| Humanities and Social Sciences | -0.937 | 0.418 | -0.031 | [-2.098,0.224] | 0.089 |
| Program Type(Bachelor’s Degree=1) | 1.458 | 0.426 | 0.052 | [0.276,2.640] | 0.027 |
| Gender（Male=1） | 0.012 | 0.184 | 0.005 | [0.499,0.522] | 0.952 |
| Place of Origin（Rural=1） | -0.303 | 0.108 | -0.009 | [-0.604,-0.002] | 0.049 |
| Family Structure（Single-parent family=1） | -2.016 | 0.247 | -0.046 | [-2.702,-1.330] | 0.001 |
| Model 2 |  |  |  |  |  |
| Major(reference group: Other) |  |  |  |  |  |
| Medical Sciences | -0.992 | 0.533 | -0.043 | [-2.470,0.487] | 0.136 |
| Natural Sciences | -1.698 | 0.095 | -0.039 | [-1.960,-1.435] | ＜0.001 |
| Engineering | -0.423 | 0.109 | -0.015 | [-0.726,-0.120] | 0.018 |
| Humanities and Social Sciences | -1.115 | 0.416 | -0.038 | [-2.270,0.041] | 0.055 |
| Program Type(Bachelor’s Degree=1) | 0.993 | 0.376 | 0.036 | [-0.051,2.036] | 0.058 |
| Gender（Male=1） | -0.105 | 0.22 | -0.002 | [-0.717,0.507] | 0.659 |
| Place of Origin（Rural=1） | 0.272 | 0.268 | 0.011 | [-0.471,1.015] | 0.367 |
| Family Structure（Single-parent family=1） | -0.418 | 0.109 | -0.01 | [-0.719,-0.116] | 0.018 |
| Family Support | 2.404 | 0.024 | 0.511 | [2.337,2.471] | ＜0.001 |
| Model 3 |  |  |  |  |  |
| Major(reference group: Other) |  |  |  |  |  |
| Medical Sciences | -0.792 | 0.352 | -0.032 | [-1.769,0.185] | 0.088 |
| Natural Sciences | -1.062 | 0.298 | -0.024 | [-1.889,-0.236] | 0.023 |
| Engineering | -0.317 | 0.282 | -0.012 | [-1.098,0.465] | 0.323 |
| Humanities and Social Sciences | -0.523 | 0.115 | -0.018 | [-0.843,-0.202] | 0.011 |
| Program Type(Bachelor’s Degree=1) | 0.096 | 0.115 | 0.001 | [-0.222,0.414] | 0.449 |
| Gender（Male=1） | -1.736 | 0.101 | -0.07 | [-2.017,-1.456] | ＜0.001 |
| Place of Origin（Rural=1） | 0.563 | 0.09 | 0.021 | [0.312,0.814] | 0.003 |
| Family Structure（Single-parent family=1） | -0.364 | 0.153 | -0.009 | [-0.789,0.060] | 0.076 |
| Family Support | 0.873 | 0.086 | 0.185 | [0.634,1.112] | 0.001 |
| School Support | 0.566 | 0.095 | 0.11 | [0.303,0.830] | 0.004 |
| Psychological Capital | 0.986 | 0.033 | 0.567 | [0.895,1.076] | ＜0.001 |
