# Supplementary material for: The association between family support and college students’ well-being: an analysis of mediating pathways
Source: Front Psychol. 2026 Sep 18;17:1860654. doi: 10.3389/fpsyg.2026.1860654 (PMC13630776; doi:10.3389/fpsyg.2026.1860654)
Supplement: Supplementary file 2 [file Data_Sheet_2.docx]

| **Supplementary Material S2: Goodness-of-fit test for the structural equation model before modification.** | | | | | | | | | | | | |
| --- | --- | --- | --- | --- | --- | --- | --- | --- | --- | --- | --- | --- |
| Indicator | χ² | df | χ²/df | GFI | AGFI | NFI | RFI | IFI | TFI | CFI | RMSEA | RMSEA 90% CI |
| Recommended criteria | — | — | <5(good) | >0.9 | >0.9 | >0.9 | >0.9 | >0.9 | >0.9 | >0.9 | <0.05 | — |
| Obtained values | 2814.151 | 125 | 22.513 | 0.971 | 0.96 | 0.981 | 0.976 | 0.981 | 0.977 | 0.981 | 0.044 | 0.043-0.046 |
